# A Population-Based 3D Atlas of the Pathological Lumbar Spine Segment

**DOI:** 10.3390/bioengineering9080408

**Published:** 2022-08-22

**Authors:** Vincenza Sciortino, Salvatore Pasta, Tommaso Ingrassia, Donatella Cerniglia

**Affiliations:** 1 Department of Engineering, University of Palermo, Viale delle Scienze, 90128 Palermo, Italy; 2 Department of Research, IRCCS-ISMETT, 90100 Palermo, Italy

**Keywords:** spinal column, pathological lumbar spine segment, SSM, PCA, biomechanics

## Abstract

The spine is the load-bearing structure of human beings and may present several disorders, with low back pain the most frequent problem during human life. Signs of a spine disorder or disease vary depending on the location and type of the spine condition. Therefore, we aim to develop a probabilistic atlas of the lumbar spine segment using statistical shape modeling (SSM) and then explore the variability of spine geometry using principal component analysis (PCA). Using computed tomography (CT), the human spine was reconstructed for 24 patients with spine disorders and then the mean shape was deformed upon specific boundaries (e.g., by ±3 or ±1.5 standard deviation). Results demonstrated that principal shape modes are associated with specific morphological features of the spine segment such as Cobb’s angle, lordosis degree, spine width and height. The lumbar spine atlas here developed has evinced the potential of SSM to investigate the association between shape and morphological parameters, with the goal of developing new treatments for the management of patients with spine disorders.

## 1. Introduction

The spine is the supporting structure of the human being. This part of the skeletal muscle system is extremely strong to ensure erect standing, support the weight of the upper body and torso, and transfer the load to the lower legs [1]. Under specific conditions or with aging, the spine can develop several pathologies, such as herniation of intervertebral disc [2] and scoliosis, as characterized by changes in Cobb’s angle [3]. Spinal biomechanics is remarkably complex due to the follower load generated by muscular trunk action, ensuring human spine stability [4,5,6]. The spinal stability is influenced by several factors, which ensure the mechanical and physiological balance of the structure. There exists a direct link between spine biomechanics and the development of a pathological condition. Any anatomical or functional alteration from the normal physiological condition is reflected in a biomechanical disorder of the main components of vertebral structure, and this ultimately leads to the occurrence of complications such as back pain [7,8,9]. A herniated or protruded disc corresponds to a crushing of the intervertebral disc, leading to a reduction in the main shock-absorbing function. If one disc fails to act, the normal functioning of the other discs is affected as well. Thus, the spine works harder and suffers from a higher stress state [10,11]. A scoliotic pathology has an even more degenerative and severe effect than herniation as this compromises the entire spinal structure [12,13,14].

Describing the shape of an anatomy as complicated as the lumbar spine is difficult. Statistical shape modeling (SSM) is a method of medical imaging analysis that allows us to visualize and quantify the variability of a given anatomy, including unique geometrical features in a patient population [15,16,17]. The SSM method applies the Principal Component Analysis (PCA), a statistical tool through which it is possible to reduce the variables of a population and cluster them within different Shape Modes [18,19,20]. This approach was used to develop virtual atlases of the anatomical region of interest [21,22,23]. In the setting of spine biomechanics, SSM models revealed the associations between demographic data and unique features of the lumbar spine [24,25,26,27,28,29].

The paper aims to develop an SSM model of the lumbar spine under pathological conditions. Two SSM models were developed, one for the vertebral body and one for the intervertebral disc. Shape Modes were obtained by deforming the mean template and then correlated to CT-based metrics using Pearson correlation. The association between specific shape modes and the anatomic variables was discussed. The mean shape represents the average spine anatomy of all patients included in the study. The shape modes are obtained by deforming the mean shapes to generate new spine geometry, highlighting the main shape changes (i.e., variance) around the mean shape model.

## 2. Materials and Methods

### 2.1. Patient Study Population

A patient study population of 24 patients underwent CT imaging for the assessment of the disease status. Specifically, a 64 detector rows CT scanner (VCT 64; GE Medical Systems, Milwaukee, WI, USA) was used to perform a total body acquisition of each patient. The scans were performed with a spiral pitch of 0.984, gantry rotation velocity of 0.5 m/s, tube voltage of 120 kV and slice thickness of 0.625 mm. For each patient, the lumbar spine from L1 to L5 was analyzed, including the intervertebral discs. Patients presented different scoliosis and herniated disc pathological conditions. For each patient, demographic data and CT-based anatomical parameters were measured (Table 1). Though all patients had one protrusion or herniated disc, 54.2% of patients had scoliosis degrees ranging from 5° to 16.74°. All imaging and clinical data were subjected to internal review board approval and patients signed informed consent prior to enrollment.

### 2.2. Lumbar Spine Segmentation

For each patient, the CT scans were segmented using the medical imaging software, Mimics v21 (Materialise, Leuven, Belgium). Specifically, the reconstruction of the lumbar spine from L1 to L5 started with semi-automatic thresholding of the spine grey values followed by manual mask editing to remove artifacts. Smoothing was performed on the segmented 3D model using 20 iterations and a smooth factor of 0.18 and was followed by wrapping with the smallest detail of 0.5 mm and a gap closing distance of 0.8 mm. A similar approach was used to segment the intervertebral discs using different thresholding and mask colors.

### 2.3. Geometrical and Anatomical Measurements

The main morphological parameters of the vertebral body and intervertebral discs were measured to quantify the severity of lordosis and scoliosis, the spine surface, perimeter, height, and width [30]. Specifically, we measured the upper and lower surface layers of the intervertebral disc in the axial plane to obtain the area, perimeter, and centroid. Therefore, we used the centroid’s position to measure the height and width of the disc and vertebral body in the coronal plane. All measurements were taken for all 24 patients. Table 2 and Table 3 show the geometrical parameter measurements for the discs and the vertebral body, respectively.

### 2.4. Pathological Issues

Several parameters were quantified to characterize the disease condition: (1) Cobb’s angle as a measure of the scoliosis degree; (2) the lordosis degree as an index of physiological or non-physiological curvature in the sagittal plane; (3) the presence of disc herniation or protrusions. The patient study population was divided into two groups: a first group of 11 patients with herniated discs, and a second group of 13 patients with herniated discs and scoliosis. Within each group, there were four patients with hyperlordosis, hence they were evenly distributed between the groups. The measurements of lordosis degree were performed by Cobb’s method, i.e., by finding the angle between the perpendicular line to the upper plate of L1 and the perpendicular line to the lower plate of L5, in the sagittal plane. A physiological degree of lordosis is between 35° and 55°, above that there is hyperlordosis, below that there is hypolordosis [31,32,33]. The measurements of the scoliosis degree were performed by Cobb’s method, i.e., by finding the angle between the tangent line to the more inclined upper plate and the tangent lie to the lower inclined lower plate. A physiological Cobb’s Angle is less than 5°, above that there is scoliosis, which might be severe depending on the angle [34]. The presence of herniation or protrusion were made from the CT images, based on a simple visualization and evaluation of them: all patients presents at least one protrusion or herniatic discs.

### 2.5. SSM Approach

The SSM was determined using a script developed in the mathematical language program MATLAB (R2020, Math-Works Inc., Natick, MA, USA) as previously described by our group [35,36]. Specifically, the lumbar spine models were resampled at sufficient resolution to quantify all shape features. Then, the iterative closest-point algorithm was used to move and align each sampled point of the lumbar spine with respect to a reference patient model (which can be observed in the appendix with its geometric measurements for the disc and vertebral body). The reference patient model is Patient n°18, the patient representative of the average population shape. Alignment was carried out by transformations minimizing the overall distance among pairs model and was repeated until the template shape significantly reduced its bias to the initial reference shape. The PCA techniques were used to reduce the complex spine shape to a few components, and this was performed using the build-in function implemented in MATLAB. Using orthogonal transformations, the PCA project the data onto a linear space of maximum variation directions, known as “shape mode” or “mode”. Shape modes are specific aspects of the anatomical variation of the vertebral body and its disc and are adopted to identify key geometrical features that cannot be described by the anatomy alone. After PCA, the number of retained modes is generally well below the number of original variables yet retains a high percentage of the overall variability in the original set. The first shape mode shows the main change in the variability of the data set while each succeeding mode has the highest residual variance possible, thereby showing specific anatomical features of the lumbar spine shape. The coordinates of the spine model are then concatenated into a shape vector and assembled into a matrix. The eigenvectors of the covariance matrix formed the principal component modes, and their corresponding eigenvalues indicated the proportion of the total variance explained by each shape mode. The contribution of each mode can be visualized deforming the template from low −3σ to high +3σ values of each mode’s deformation vector. Shape vectors numerically represent the contribution that each shape mode has on each spine model [17,18,19,20,21,22].

## 3. Results

**Figure 1:**
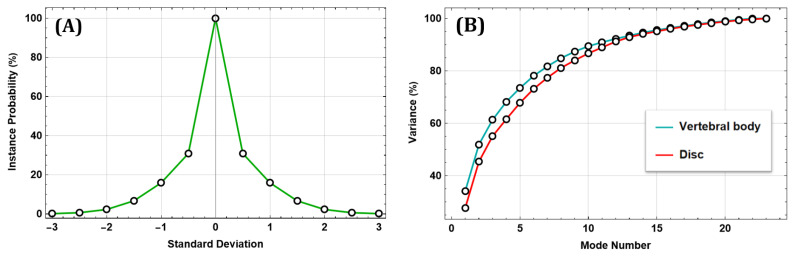
SSM model: (**A**) Instance probability vs Standard Deviation. (**B**) Variance vs Mode Number.

The realized SSM allows us to obtain a digital population of the pathological lumbar spine. Figure 1A shows the profile of the instance probability. The latter underlines a Gaussian trend and represents the chance that the specific deformed shape occurs for a given value of the shape boundary. This curve shows that, for shape deviations of 0.5σ, 1σ, 1.5σ, 2σ, 2.5σ, 3σ, the deformed shape probability is, respectively, of 30.85%, 15.87%, 6.68%, 2.27%, 0.62% and 0.13%. Figure 1B displays the scree plot with the cumulative variance, reaching 100% as the Mode number increases. The PCA model shows that the first 12 Shape Modes catch the 90% shape variability.

**Figure 2:**
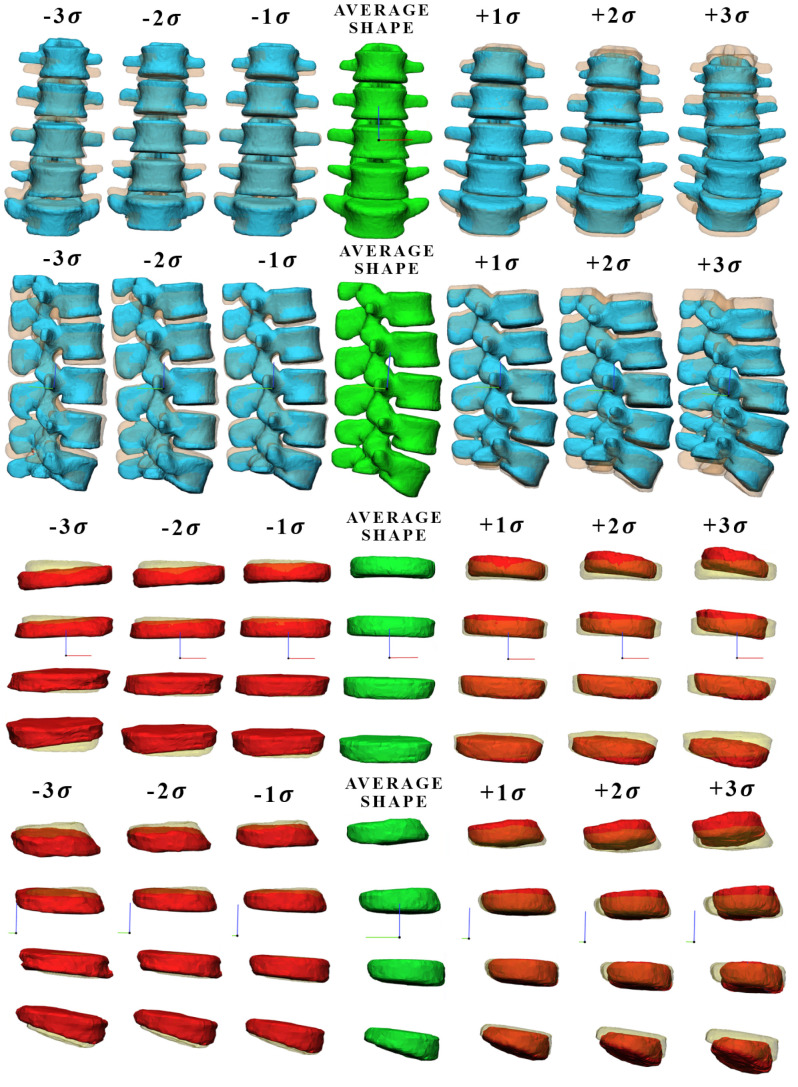
Graphic representation of the vertebral body and intervertebral disc for shape mode 1 at different values of σ, where each deformed shape mode was overlapped with the mean shape (transparent shape below).

**Figure 3:**
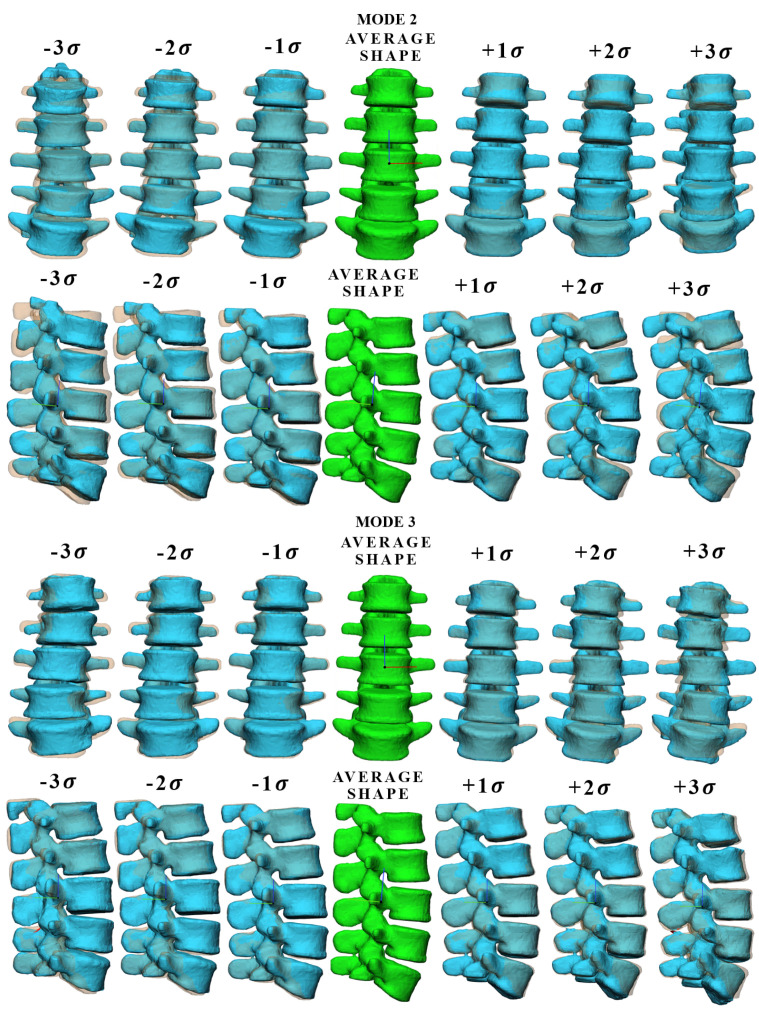
Graphic representation of vertebral body for shape mode 2 and mode 3 at different values of σ, where each deformed shape mode was overlapped with the mean shape (transparent shape below).

**Figure A1:**
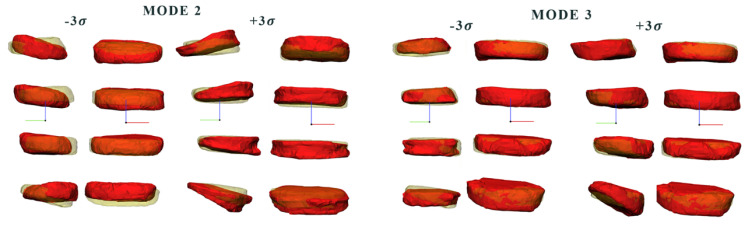
Graphic representation for mode 2 and 3 for the intervertebral disc at different values of σ, where each deformed shape mode was overlapped with the mean shape (transparent shape below).

Figure 2 shows mode 1 of the vertebral body and intervertebral disc, at different levels of standard deviation ( ±1σ, ±2σ, ±3σ), where each deformed shape mode was overlapped with the mean shape of the model. For the vertebral body, mode 1 accounts for the 34% of the total variance in our patient study group and is mainly associated with a proportional change (scale factor) of the spinal height. For the intervertebral disc, mode 1 accounts for 27% of the total variance in our patient study group and is mainly associated with a variation of surface, width, and scoliosis degree. Figure 3 shows mode 2 and mode 3 of the vertebral body, which account for 52% and 61% of the total variance, respectively. As the standard deviation changes, the lumbar degree (mode 2) and scoliosis degree (mode 3) vary significantly. Mode 2 and mode 3 (Figure A1) of the intervertebral disc are shown in the Appendix A, they account for 45% and 55% of the total variance and are mainly associated with different multiple geometric aspects, each of them.

Videos S1 and S2 display the animation of mode 1 for the vertebral body and intervertebral disc, respectively. Video S3 displays an animation of mode 2 and mode 3 for the vertebral body.

### 3.1. Comparison between Modes

**Figure 4:**
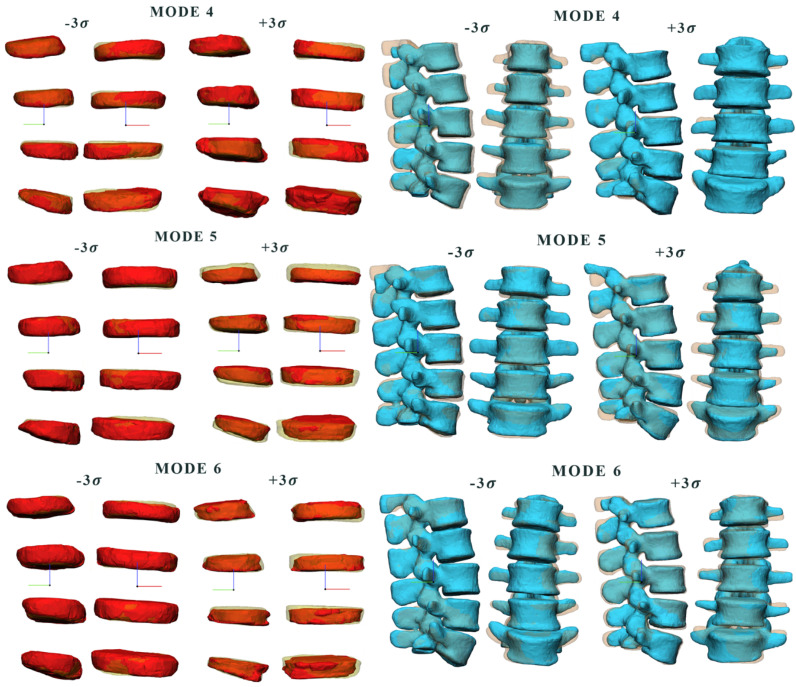
Graphic representation of the vertebral body and intervertebral disc for shape mode 4, 5, 6 at different values of σ, where each deformed shape mode was overlapped with the mean shape (transparent shape below).

Figure 4 shows the different shape modes for the intervertebral disc and the vertebral body, in the frontal and lateral views, where each deformed shape mode overlapped with the mean shape of the model. For the intervertebral disc, we displayed modes 4, 5 and 6, which account for 61%, 67% and 73% of the total variance, respectively, and are associated with variations of lumbar degree (mode 4), surface (mode 4, 5 and 6), width (mode 4, 5 and 6), height (mode 5 and 6), scoliosis degree (mode 4, 5 and 6), and individual heights of single discs (mode 6). For the vertebral body, we displayed modes 4, 5 and 6 which account for 68%, 73% and 78% of the total variance, respectively, and are associated with variations of width (mode 4), height (mode 4 and 6), and spinal process dimensions (mode 4, 5 and 6).

### 3.2. Relationship between Anatomical Aspects and Shape Modes

The last step involved checking whether there were any correlations between the identified shape modes, and anatomical and pathological aspects of patients. The latter are considered in looking for correlations including: lumbar spine height, lordosis degree, Scoliosis degree, mean perimeter, patient height, BMI, weight, and mean width.

Firstly, both groups created were required to identify any links between the measured anatomical, morphological, and pathological data and the relative p-value, which was obtained from a statistical t-test. The lumbar spine height was used as an index of height variation due to the presence of a hernia or protrusion. A direct link between the patient’s weight and the herniated disc were identified (*p* = 0.050), as well as between weight and the scoliosis degree (*p* = 0.110).

**Figure 5:**
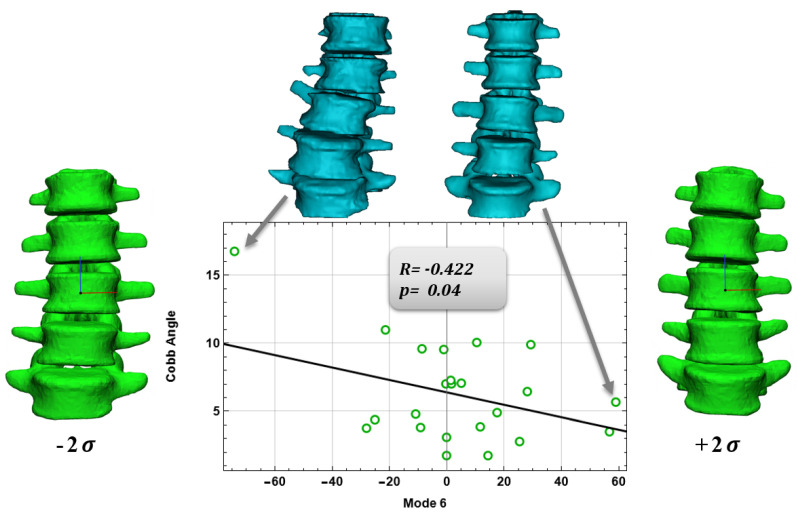
Correlation for the vertebral body of mode 6 and Cobb’s Angle.

**Figure 6:**
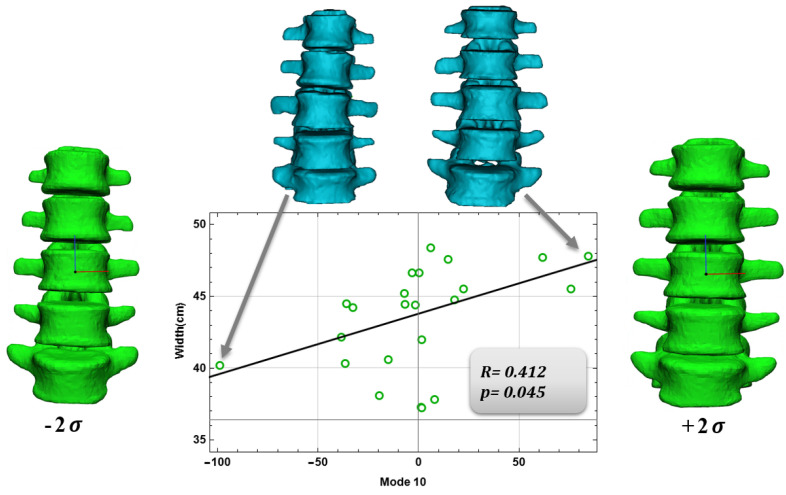
Correlation for the vertebral body of mode 10 and Width.

**Figure 7:**
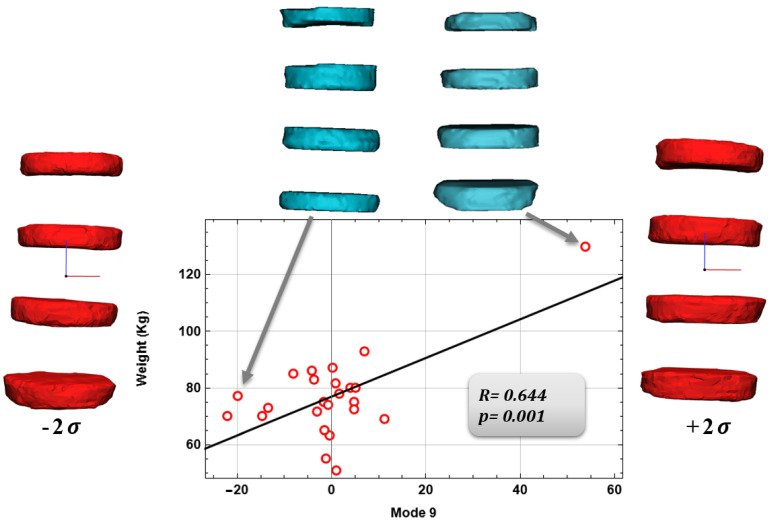
Correlation for the intervertebral disc of mode 9 and Weight.

For the vertebral body, a negative correlation was found between mode 6 and Cobb’s angle (*p* = 0.04, R = –0.422, Figure 5) and a positive correlation between Mode 10 and mean column width (*p* = 0.045, R = 0.412, Figure 6). Finally, for the intervertebral disc a positive correlation was found between mode 9 and patient weight (*p* = 0.001, R = 0.644, Figure 7). Vertebral body modes 6 and 9 account for 78% and 87% of the cumulative variance of geometrical shape, respectively, and represent variations in terms of scoliosis degree (mode 6) and vertebral body width (mode 9). Intervertebral disc Mode 10 accounts for 84% of the cumulative variance of geometrical shape, and represents variations in terms of intervertebral disc height and so on of the herniated disc.

Figure 5, Figure 6 and Figure 7 show the graphs between the modes and their respective comparison parameters, with the trend line, the patients’ extremes, and the changes in mode shape corresponding ±2σ.

## 4. Discussion

The present study shows an SSM of the lumbar spine segment of the human body under pathological conditions. The atlas has allowed us to extrapolate several shape modes that visually and numerically describe the complex shape of the lumbar spine to a level of details previously impossible with conventional imaging modalities. The shape modes were analyzed to visually derive unique geometrical changes in the lumbar spine, and then the extracted shape modes by PCA were statistically correlated to biomechanical variables to shed light on shape and function. The SSM here proposed has demonstrated the potential of shape analysis for discovering previously unknown geometrical features that may improve the way we diagnose and treat the diseased lumbar spine. Moreover, the SSM allows us to obtain new lumbar spine models to develop computational analyses to assess spine biomechanics. However, future studies are needed to confirm the potential of the proposed 3D atlas of the lumbar spine segment in a large patient population.

For the 24 patients, the spine segment was segmented, and a series of measurements were performed to determine the main geometrical and morphological features of anatomical regions of interest, i.e., surface, height, width, and perimeter. Some correspondence concerning the numerical values of the measurements made by Divya et al. [30] was observed at the patient geometry measurements, thus validating the approach we used to make the same measurements. Therefore, the SSM models for the vertebral body and intervertebral disc were created [28,30].

First, a statistical description of the anatomical and pathological data was conducted by separating the population into two groups, through a statistical t-test. We observed how weight and, so, BMI have an important influence on the presence or absence of herniated discs or scoliosis. As weight and BMI increase there is a greater likelihood of an advanced degree of scoliosis or herniated pathology. However, these pathologies depend on many factors, which are not purely quantitative but also qualitative, relating to the personal lifestyle, posture during the daily routine and many other factors. This confirms the studies of Brown et al. [37] who performed a descriptive statistics analysis to understand how anatomical data influences the presence of spinal pathologies, so we have, as described in this study.

**Figure A2:**
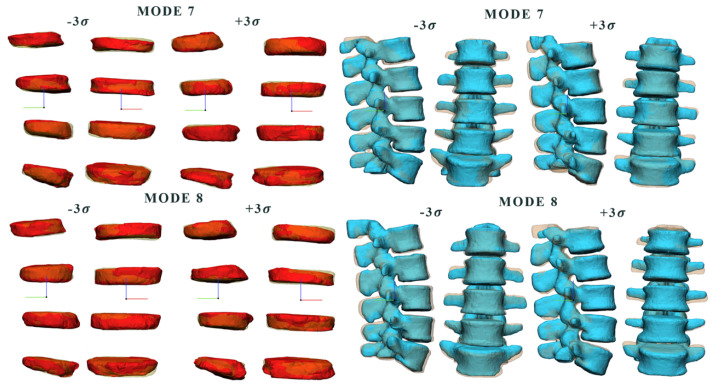
Graphic representation for modes 7 and 8 for intervertebral disc and vertebral body at different values of σ where each deformed shape mode was overlapped with the mean shape (transparent shape below).

**Figure A3:**
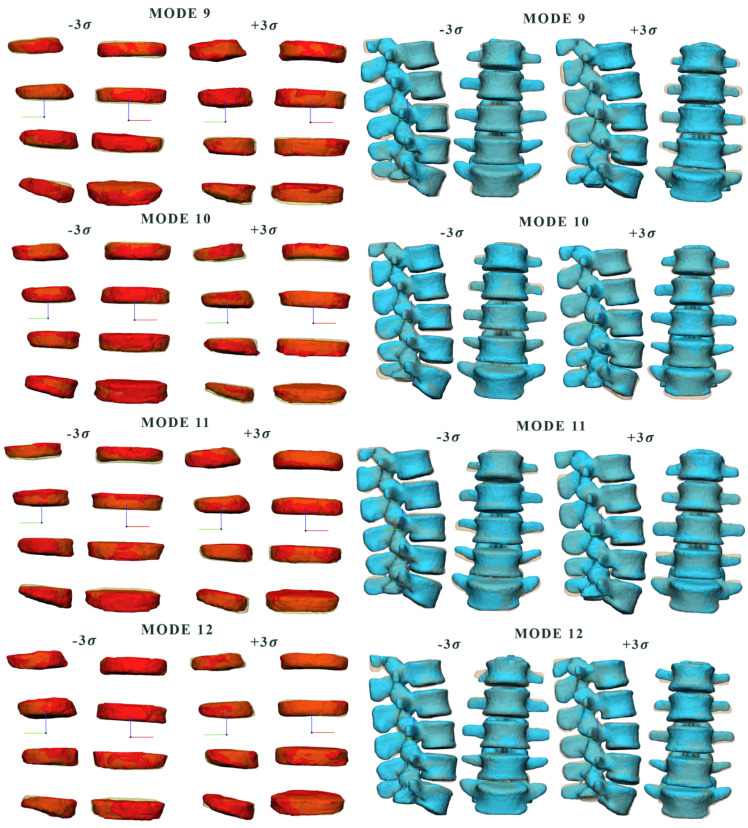
Graphic representation for modes 9, 10, 11 and 12 of the vertebral body and intervertebral disc at different values of σ, where each deformed shape mode was overlapped with the mean shape (transparent shape below).

**Figure A4:**
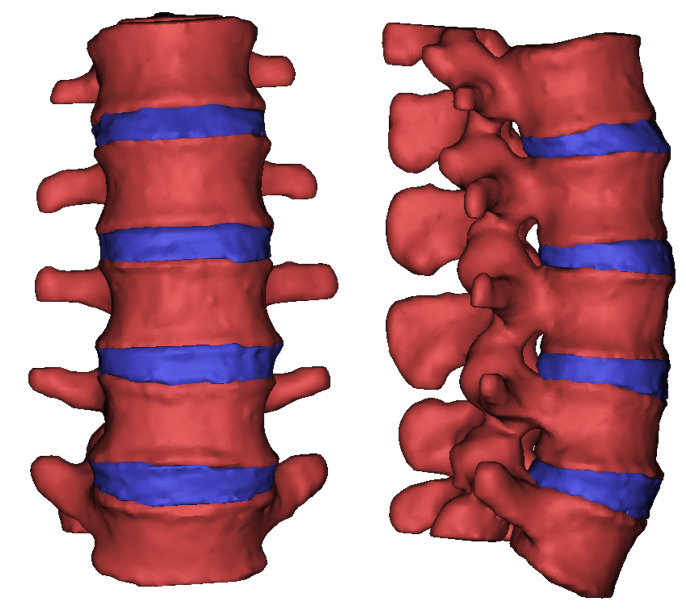
Reference patient model segmentation used to realize the SSM model.

Therefore, the different Shape Modes obtained by the principal component analysis (PCA) instrument were determined. These Modes allow us to observe the geometrical and morphological variation of these anatomical regions, as the standard deviation changes. Frontal and lateral views of the vertebral body and intervertebral disc geometries were observed to provide better visualization and understanding of which parameters are related to the changes. The first 12 modes account for the largest variations in pattern shape, as much as 90%. Firstly, the ±3σ variations of the first three modes of the vertebral body and mode 1 of the intervertebral disc were analyzed. For the disc, mode 1 changes the scoliosis degree, surface, and width. On the other hand, for the vertebral body, there is a variation in height, lordosis degree and Cobb’s Angle. The remaining modes, 7 to 12, are shown in the appendix (Figure A2, Figure A3 and Figure A4).

Bibby et al. [38] have found that scoliosis plays an important role in the degenerative process of the intervertebral disc, deteriorating the disc end-plate and its permeability. Our study allowed us to identify from the SSM model how effectively a variation in the scoliosis degree significantly affects the disc height, which is a symptom of crushing and therefore the onset of herniated pathology. The presence of a herniated disc leads to a decrease in the column spine height, because of the crushing of the intervertebral disc, resulting in a disc protrusion or rupture. Therefore, the herniation affects the surface of the intervertebral disc and, consequently, changes the width of the disc itself, resulting in a more flattened disc. Scoliotic pathology is determined by a change in the Cobb angle of the spine in the frontal (coronal) plane. More than half of the patients have an advanced degree of scoliosis, with a Cobb’s Angle greater than 5°. Scoliosis occurs by changing the frontal rectilinear physiological nature of the spine, with a certain curvature. This curvature reflects in the height of the intervertebral disc and surface. Often, a scoliotic pathology is accompanied by a herniated pathology, as by varying its curve, the normal biomechanics of the spine is altered. The intervertebral disc is subjected to increased stress, which deteriorates it, leading to crushing, protrusions, and herniation, as shown by several studies [39,40]. From the segmentation of the images and the scoliosis angle measurements taken, we found that patients who had a high scoliosis angle have also at least one herniated disc. This further confirms the direct link between herniated and scoliotic pathology.

Finally, Pearson correlations were found between the Shape Modes, and anatomical and physiological characteristics. Several authors have made SSM models of the spine considering, for example, healthy patients, but also spine deformities. Dai, J. [28] and Yong, R. [29], and their collaborators, limited their work to just the variation of the spinal geometry and deformity, they have not actually considered, as we have with our work, the possible correlations that we have found between the shape modes and the anatomical and pathological features. Once, we found significant Pearson correlations between mode 6 and Cobb’s angle, mode 10 and mean column width for the vertebral body; for the intervertebral disc, a positive correlation was found between mode 9 and patient weight. These correlations represent the actual link between the shape modes and the anatomical and pathological characteristics of the anatomical region in question.

The study has several limitations that are mainly caused by the limited number of patients included in the SSM. Indeed, SSM could be more powerful when a large patient population is considered. This can also allow us to stratify patients into more uniform groups than that here proposed. The stratification permits us to obtain a virtual population that includes all the possible pathological situations of the human spine. This model could represent an individual shape mode for the single variation of geometry, anatomy, and pathology, without mixed conditions, as observed in this study. A control study group of patients without spine disorders was not included in this SSM analysis. Indeed, the development of an atlas for the healthy spine and the comparison with the current diseased atlas can shed light on whether the spine deformation around the mean templates is associated with the disorders and/or the expected variance of the adult population. In future studies, the present approach will be expanded to a large patient population to confirm the present findings.

## 5. Conclusions

The present study has allowed us to obtain a virtual atlas of the spinal lumbar segment, to assess the associations between shape and function. This SSM can also be used to generate a new model by deforming the mean template of the lumbar spine to specific boundaries. In future studies, the present SSM will be strengthened by a large patient cohort and will then be used to generate new anatomies for the assessment of the biomechanical response of the human spine to variations in the shape features.

## Figures and Tables

**Table 1:** Clinical characteristics of patient study population.

Characteristics	Patients
Age (Years)	55.9 ±7.5
Male (%)	79.2
Weight (Kg)	76.9 ±7.5
Height (m)	1.70 ±0.01
BMI ^1^	26.6 ±4.3
BSA ^1^	1.90 ±0.2
Herniatic Disc (%)	91.7
Scoliosis (%)	54.2

^1^ Note: BSA = Body Surface Area; BMI = Body Mass Index.

**Table 2:** Medium Measurements of 24 patients’ disc.

Medium Shape	Medium Surface (mm${}^{2}$)	Height (mm)	Medium Perimeter (mm)
L1-L2	1470.21 ±256.1	10.01 ±2.2	147.56 ±11.3
L2-L3	1964.22 ±287.8	10.84 ±2.1	151.19 ±12.3
L3-L4	1629.95 ±258.9	11.28 ±2.3	152.85 ±11.6
L4-L5	1401.37 ±284.3	10.30 ±2.6	149.47 ±12.3

**Table 3:** Medium Measurements of 24 patients’ vertebral body.

Medium Shape	Medium Surface (mm${}^{2}$)	Height (mm)	Medium Perimeter (mm)	Width (mm)
L1	1407.3 ±215.9	27.8 ±1.9	143.9 ±9.9	40.4 ±3.4
L2	1473.9 ±222.8	27.7 ±2.2	145.2 ±10.6	41.7 ±3.8
L3	1555.9 ±230.4	28.1 ±1.8	148.4 ±10.6	43.8 ±3.9
L4	1534.6 ±222.8	27.7 ±2.2	149.2 ±10.6	44.7 ±3.8
L5	1479.5 ±198.8	30.1 ±3.1	149.0 ±9.5	45.2 ±7.4

## Data Availability

The data presented in this study are available on request from the corresponding author. The data are not publicly available due to ethical reason.

## Supplementary Materials

The following supporting information can be downloaded at: https://www.mdpi.com/article/10.3390/bioengineering9080408/s1, Video S1: Mode 1 for lumbar vertebral body. Video S2: Mode 1 for intervertebral disc. Video S3: Mode 2 and Mode 3 for lumbar vertebral body.

## Appendix A

Other Shape Modes of vertebral body intervertebral disc are shown, and the reference patient of the model is shown, with its geometrical measurements.

**Table A1:** Measurements of reference model patient of the intervertebral disc.

Medium Shape (mm${}^{2}$)	Medium Surface (mm)	Height (mm)	Medium Perimeter( mm)
L1-L2	1395.88	9.99	152.17
L2-L3	1732.14	9.99	155.08
L3-L4	1733.20	8.24	156.51
L4-L5	1846.57	10.00	159.95

**Table A2:** Measurements of reference model patient of the vertebral body.

Medium Shape (mm${}^{2}$)	Medium Surface (mm)	Height	Medium Perimeter (mm)	Width (mm)
L1	1556.57	27.97	152.07	45.13
L2	1644.28	26.13	152.51	47.50
L3	1705.84	28.06	159.51	46.62
L4	1600.47	26.22	151.27	47.48
L5	1528.99	28.20	153.79	48.34
